# Handrail support interference in cardiac autonomic modulation adjustments in young adults during maximal exercise testing

**DOI:** 10.1038/s41598-020-68155-3

**Published:** 2020-07-08

**Authors:** Giovanna Lima de Oliveira, Adriana Hernandez Marques, Vanessa Ferrari da Fonseca, Beatriz Augusta Pozzolo, Fernanda Panacioni, Taís Capucho Santos, Amanda Archeleiga Guedes, Aurenzo Gonçalves Mocelin, Renata Labronici Bertin, Anderson Zampier Ulbrich

**Affiliations:** 1 Research Group of Exercise Medicine (MedEx), Federal University of Parana (UFPR), Padre Camargo Street, 280 – Alto da Gloria, Curitiba, PR 80060-240 Brazil; 2 Department of Nutrition, Center for Health Sciences, Federal University of Parana (UFPR), Curitiba, PR Brazil; 3 Department of Integrative Medicine, Center for Health Sciences, Federal University of Parana (UFPR), Curitiba, PR Brazil

**Keywords:** Physiology, Cardiology

## Abstract

The aim of this study was to investigate whether the use of handrail support during maximal exercise treadmill testing (ETT) would interfere in cardiac autonomic modulation kinetics when compared to not using handrail support. The hypothesis of overestimation in cardiac autonomic dynamics when the ETT is performed using handrail was tested. Thirty-five undergraduates (21.08 ± 2.98 years old) of both sexes, volunteered to undertake two ETT under the *Ellestad* protocol, in non-consecutive days. The first test (T1) was performed with handrail support and, after 7 days, the second test was performed (T2) without the support. Autonomic function was measured by heart rate variability (HRV) during both tests and resting. Estimated value of peak oxygen uptake (VO_2_) was 22.4% (p < 0.0001) higher in T1 when compared to T2. Overall, parasympathetic pathway was deactivated earlier in T2 than in T1, with NNxx measures variating in T1 from 10.74 ± 14.59 (ms) and in T2 from 3.48 ± 3.79 (ms). In stage two, mean values of HF in T2 corresponded to 32% of values in T1. Stage three presented a difference of 60% (p < 0.014) in LF between means reached in T1 and T2. Lastly, the association of LF and VO_2_ persisted longer in T1 stages than in T2 and was verified in early stages (S2 and S3) of both ETTs. Our findings suggest that parasympathetic influences on HR were slightly prolonged during ETT when subjects hold onto the treadmill.

## Introduction

Physical exercises are an important protective factor for cardiovascular diseases^1–3^. In addition to modifying the metabolism, regular exercise allows greater balance in the autonomic modulation of the cardiovascular system, increasing parasympathetic activity and decreasing sympathetic activity^1,4–6^. The cardiac autonomic control network processes stimuli from baro- and chemoreceptors and generates a reflex response responsible for heart rate (HR) adjustments^1^. Studies indicate that active and fit individuals have greater cardiac responsiveness to autonomic control when compared to sedentary individuals^2,5,7,8^.

Thus, the evaluation of the autonomic modulation of the cardiovascular system may indicate parameters of an individual’s lifestyle^3^. Heart rate variability (HRV) is a noninvasive marker used to determine the analysis of the two autonomic nervous system pathways^2,6^. Previous studies have demonstrated the post-exercise delay in the recovery of HRV to be a predictor of mortality in heart failure patients^1,9,10^. Nevertheless, an increase in HRV can be observed in individuals who exercise regularly^2,3^.

Exercise treadmill testing (ETT) is used in the evaluation of cardiovascular responses to physical stress^11^, therefore several studies have evaluated the recovery time of HRV to analyze the autonomic reactivation in different exercise intensities and modes^9,12–15^. Although few studies have evaluated the HRV response during exercise, some have shown that the high-frequency (HF) component of the HRV, which is associated with vagal activity, gradually decreases until reaching the anaerobic threshold of exercise^9,12,13^. After this, HF stabilizes. Confirming these findings, Shiraishi et al.^16^ recently presented, in real time, the HRV dynamics during an incremental ETT. They observed that the ventilatory threshold corresponds to the dynamics of the high frequency (HF) response in healthy young adults and in individuals who suffered myocardial infarction.

In the light of the above considerations, the *American Heart Association* (AHA)^11^ recommends the standardization of the execution of the stress test, especially regarding the act of holding on to the treadmill handrails. AHA emphasizes that inadequate positioning, such as holding on to the front and side handrails, can reduce body load, with subsequent reduction of physical exertion, hence underestimating physical and hemodynamic responses^11^. Considering that ETT is widely used to assess physical capacity, verify risk and diagnose cardiovascular diseases, there is a necessity to evaluate the effect of the individual’s position on hemodynamic responses. This is important for setting the best standard for implementing the ETT. Thusly, this study sought to analyze the HRV kinetics during the ETT, performed with and without handrail support by apparently healthy university students. It was hypothesized that tests that do not follow the *guidelines*^11^ would result in an overestimation of the HRV dynamics.

## Results

The anthropometric characteristics of the participants are described in Table 1. From these, 31.4% of the volunteers were above the recommended BMI and fat percentage^17^. Considering the waist circumference (WC) classification^18^, only 5.7% (n = 1) of the students might be at higher risk for cardiovascular disease.

**Table 1:** Descriptive features of subjects and cardiopulmonary and hemodynamic responses in different ETTs.

Features	N = 35
Age (years)	21.08 ± 2.98
Men, n (%)	19 (54.3)
Weight (kg)	66.66 ± 13.27
Height (cm)	169.1 ± 9.4
BMI (kg/m^2^)	23.11 ± 3.09
WC (cm)	76.5 ± 8.1
Sum DC (mm)	108.38 ± 35.1
%F (%)	18.80 ± 5.22
WHR	0.45 ± 0.03

Hemodynamic and cardiopulmonary responses	T1	T2	*p*-value
HR rest (bpm)	96 ± 12	97 ± 15	0.771
HR max (bpm)	188 ± 12	187 ± 11	0.830
SBP rest (mmHg)	117 ± 13	114 ± 13	0.257
DBP rest (mmHg)	73 ± 9	69 ± 9	0.052
SBP max (mm Hg)	166 ± 19	163 ± 22	0.498
DBP max (mm Hg)	79 ± 9	74 ± 9*	0.021
VO_2_peak (ml kg^−1^ min^−1^)	41.07 ± 7.49	33.28 ± 5.29*	< 0.001

*T1* Test 1 (holding on to the treadmill), *T2* Test 2 (not holding on to the treadmill), *BMI* Body Mass Index, *WC* waist circumference, *%F* percentage of fat, *WHR* waist height ratio, *HR* rest resting heart rate, *HRmax* maximum heart rate, *SBPrest* systolic blood pressure at rest, *DBPrest* diastolic blood pressure at rest, *SBP max* maximum systolic blood pressure in the test, *DBP* max maximum diastolic blood pressure in the test, *VO*_*2*_*peak* Maximum VO_2_.

Values reported in mean ± standard deviation; *p < 0.05.

**Figure 1:**
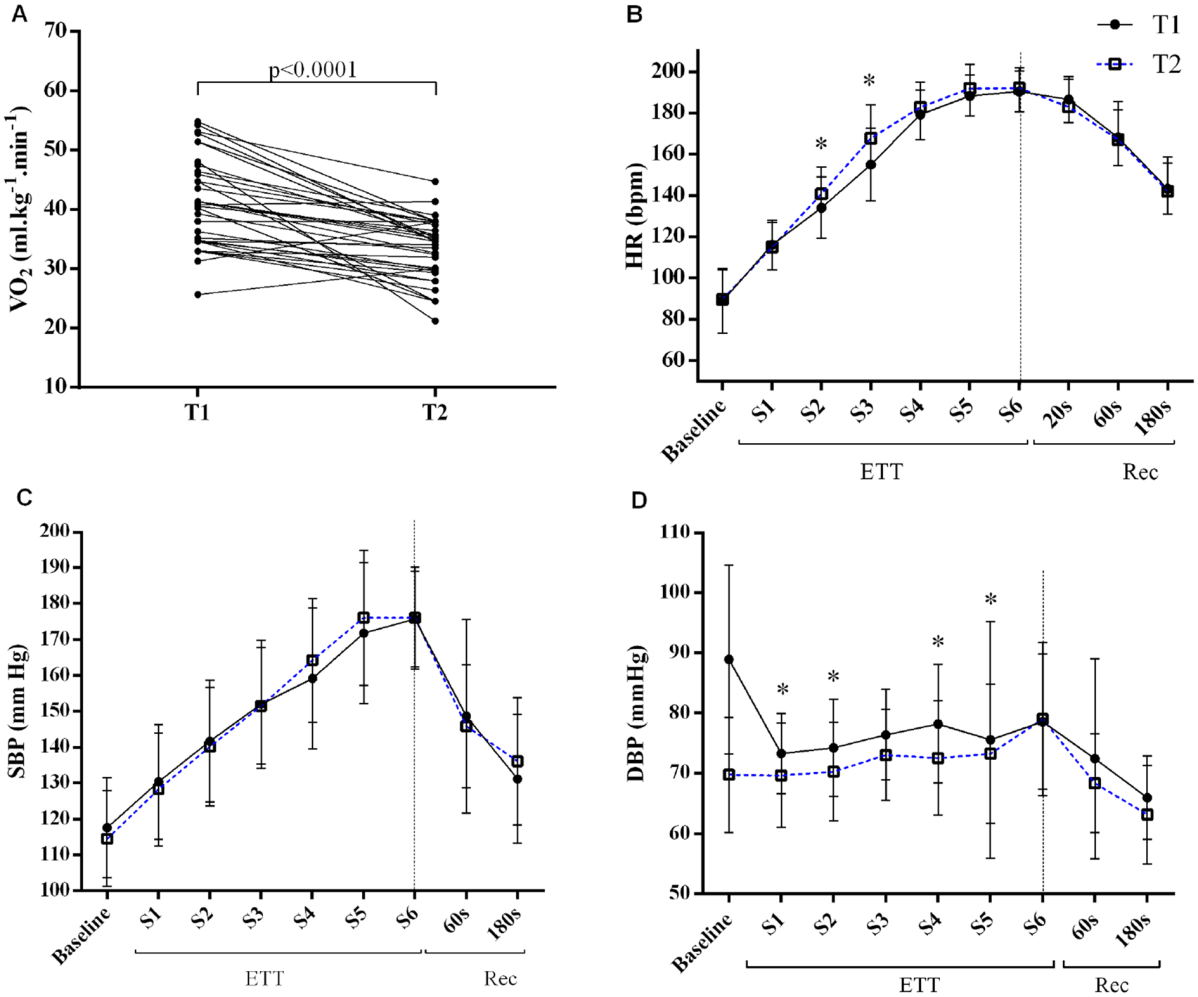
Hemodynamic and cardiopulmonary responses during different methods of ETT. (**A**) Graphical analysis of the VO_2_ for each test in T1 and T2, in which each line corresponds to a subject that connects the points representing the ETT (T1 and T2). (**B**–**D**) represent the graphs of mean HR, SBP and DBP at each stage of the T1 and T2 tests. *ETT* exercise treadmill testing stages without recovery, *REC* time interval of post-ETT recovery (3 min); Baseline rest; *S1* stage one, *S2* stage two, *S3* stage three, *S4* stage four; *S5* stage five, *S6* stage six. The values are represented by mean and standard deviation at each stage. The numbers of subjects that completed each stage for T1 and T2, respectively (S1: n = 35, 35; S2: n = 35, 35; S3: n = 35, 35; S4: n = 30, 28; S5: n = 21, 12; S6: n = 11, 1; S7: n = 3, 0). *p < 0.05: * indicates difference between groups in the specific stage.

Figure 1A shows the VO_2_ performance (rows), which revealed an estimated 22.4% lower VO_2_ (p < 0.0001) in T2 than in T1. Based on the hemodynamic response, DBP (p < 0.021) were lower in T2 (Table 1). When observed by stages, mean DBP values differed statistically (Fig. 1D) in stages one (p < 0.028), two (p < 0.024), four (p < 0.013), and five (p < 0.007), being higher in T1. The opposite occurred in the HR, in which T2 values were higher than T1 in stages 2 and 3 (p < 0.001) (Fig. 1B).

We also analyzed the mean values of the frequency and time domain components of HRV during the two tests and at rest (Table 2). We observed smaller means for the variables representing the parasympathetic system (High Frequency activity) (p < 0.050) and a number of successive RR interval pairs that differed more than “*xx”* millisecond (NNxx) (p < 0.005) in T2, when compared to T1. The same comparison was performed separately for each sex. The results did not diverge from the ones found in the general analysis (Table 2) and are presented as Supplement 1 (Table S1).

**Table 2:** Overall evaluation, disregarding the stages, of the HRV variables at rest, holding and not holding on to the handrails of the treadmill.

	Rest	T1	T2	*p*-value^#^
**Time domain**				
RR (ms)	834.8 ± 101.7	385.68 ± 71.6	369.8 ± 23.2	0.388
SDNN (ms)	51.88 ± 20.97	10.32 ± 6.26	10.15 ± 8.36	0.922
RMSSD (ms)	52.47 ± 26.53	9.34 ± 6.9	9.02 ± 7.1	0.826
NNxx (beats)	177.57 ± 106.94	10.74 ± 14.59	3.48 ± 3.79*	0.005
**Frequency domain**				
VLF (ms^2^)	176.14 ± 146.73	11.94 ± 10.18	13.14 ± 11.85	0.629
HF (ms^2^)	1,296.4 ± 1,307.8	38.28 ± 51.37	22.00 ± 27.59*	0.050
LF (ms^2^)	1,336.6 ± 1,175.06	66.05 ± 56.79	55.17 ± 48.84	0.265

*RR* RR interval, *SDNN* standard deviation of all normal RR intervals, *RMSSD* root mean square of successive squared differences between adjacent normal RRs, *NNxx* number of interval differences of successive intervals NN greater than xx = 39 40, 50 ms, *VLF* very low frequency component, *HF* high frequency component, *LF* low frequency component, *LF/HF* ratio between LF and HF.

*p < 0.05.

^#^Paired comparison between T1 and T2; values reported in mean ± standard deviation (n = 35).

**Figure 2:**
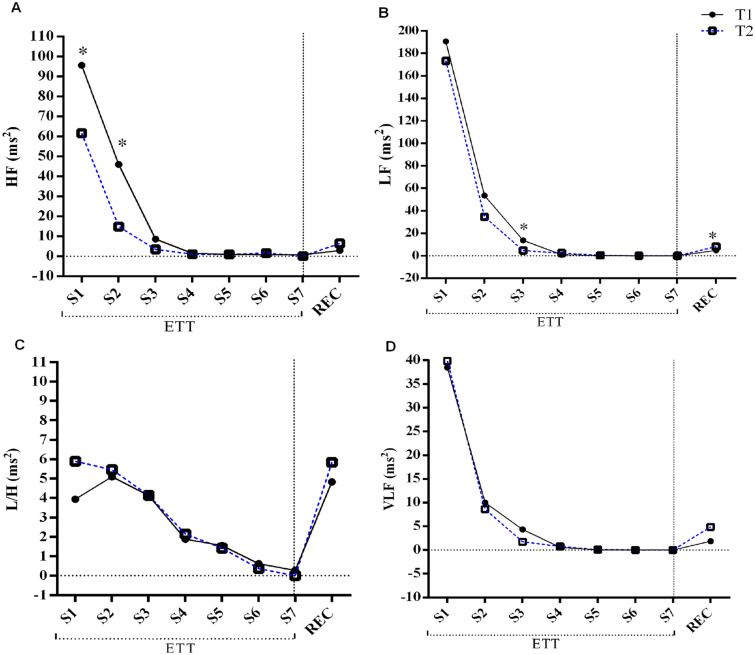
Component dynamics of HRV frequency during ETT. Graphical analysis at each stage of the tests T1 and T2 for (**A**) the high frequency component (HF), (**B**) the low frequency component, (**C**) the ratio between LF and HF, and (**D**) very low frequency. The considered values correspond to the mean of all the tests performed in each execution mode, for each stage. *ETT* exercise treadmill testing stages without recovery, *REC* to the recovery period of the test (3 min), *S1* stage one, *S2* stage two, *S3* stage three, *S4* stage four, *S5* stage five, *S6* stage six, *S7* stage seven. The values are represented by mean and standard deviation at each stage. The numbers of subjects that completed each stage for T1 and T2, respectively (S1: n = 35, 35; S2: n = 35, 35; S3: n = 35, 35; S4: n = 30, 28; S5: n = 21, 12; S6: n = 11, 1; S7: n = 3, 0). *p < 0.05. *Indicates difference between groups in the specific stage.

Figure 2 shows the modifications of the frequency components (HF, LF, VLF and LF/HF) at each stage of the ETT. There was an abrupt fall in HF after exercise on both ETTs, until the complete inhibition of parasympathetic activity (Fig. 2A). Moreover, specifically in stage 2, from the third to the fifth minute, the mean values at T2 corresponded to 32% of those measured in T1 (p < 0.048) (Fig. 2A).

Both HF and LF indexes (Fig. 2A,B) decreased their values progressively during ETT. However, LF (Fig. 2) presented a lower reduction than HF. The representative variable of baroreflex function (LF) system presented lower numbers in T2 from the beginning of the ETT up to stage 4. We observed a difference of 60% (p < 0.014) in stage 3 between the means reached in T1 and T2. During the recovery period (3 min) of ETT, there was an overlap of T2 over T1 for HF component as well as the LF and VLF. In this case, LF was 41% higher in T2 (p < 0.036).

Based on the regression analysis, independently on the sex, it was possible to determine the association of VO_2_ with HRV components in the different stages of ETT (Table 3). During T1, we observed an association of VO_2_ with LF in stages 1 (p < 0.032), in 3 (p < 0.021) and VLF in 4 (p < 0.015). In the first stage, each unit (ms^2^) that increased in the LF, had an addition of 0.013 ml kg^−1^ min^−1^ in the VO_2_ mean. This is similar to the result observed for T2, in which the increase was 0.012 ml kg^−1^ min^−1^ in VO_2_. A greater association of LF occurred in stage 2 (p < 0.011) in T2. On the other hand, in T2 recovery time, the statistically significant association (p < 0.001) was negative, meaning that there was a decrease of 0.244 ml kg^−1^ min^1^ in the mean of VO_2_, when there was an increase of one unit (ms^2^) of VLF.

**Table 3:** Association of VO_2_ with the components of HRV determined in the stages of the different ETT.

	S1 β (95% CI)	*p*-value	S2 β (95% CI)	*p*-value	S3 β (95% CI)	*P-value*	S4 β (95% CI)	*p*-value	S5 β (95% CI)	*p*-value	REC β (95% CI)	*p*-value
**T1**												
VLF	0.033 (− 0.071; 0.137)	0.524	0.094 (− 0.507; 0.695)	0.752	0.545 (− 1.453; 0.363)	0.230	3.939 (0.812; 7.066)	0.015*	− 3.355 (− 20.465; 13.754)	0.684	− 0.345 (− 1.123; 0.432)	0.372
LF	0.013 (0.001; 0.025)	0.032*	0.028 (− 0.003; 0.058)	0.073	0.139 (− 0.022; − 0.255)	0.021*	0.597 (− 2.491; 3.666)	0.698	1.548 (− 6.780; 9.875)	0.701	− 0.087 (− 0.816; 0.641)	0.808
HF	− 0.014 (− 0.053; 0.026)	0.481	− 0.017 (− 0.060; 0.026)	0.426	− 0.185 (− 0.528; 0.158)	0.281	0.424 (− 0.341; 1.189)	0.265	1.648 (0.144; 3.153)	0.033*	0.132 (− 0.118; 0.381)	0.290
**T2**												
VLF	0.021 (− 0.041; 0.083)	0.497	0.168 (− 0.506; 0.171)	0.320	1.088 (− 0.360; 2.536)	0.135	0.469 (− 0.668; 1.607)	0.404	2.031 (− 6.786; 10.848)	0.610	− 0.244 (− 0.381; − 0.107)	0.001*
LF	0.012 (0.002; 0.021)	0.020*	0.056 (0.014; 0.098)	0.011*	0.264 (− 0.713; 0.186)	0.241	− 0.612 (− 1.693; 0.469)	0.255	− 1.110 (− 4.783; 2.563)	0.511	− 0.134 (− 0.331; 0.064)	0.178
HF	− 0.016 (− 0.048; 0.015)	0.293	− 0.051 (− 0.133; 0.032)	0.219	0.108 (− 0.070; 0.285)	0.227	0.471 (− 2.062; 3.004)	0.705	0.446 (− 1.039; 1.931)	0.518	0.010 (− 0.176; 0.158)	0.906

Values reported in calculated value of β (minimum value − maximum value). The value of β corresponds to how many ml kg^−1^ min^−1^ are added to VO_2_ when a unit of the HRV variable is increased. The analysis was performed only including values from S1 to S5 because the number of subjects who participate of S6 and S7 were not sufficient to obtain statistically significant results.

*VLF* very low frequency component, *HF*: high frequency component, *LF*: low frequency component, *L/H* ratio between LF and HF, *REC* to recovery period of the test (3 min), *S1* stage one, *S2* stage two, *S3* stage three, *S4* stage four, *S5* stage five, *REC* recovery time to ST with a 3-min duration.

*p < 0.05**.**

As a supplementary analysis (Supplement S1), the same analyses were performed separately for female (Supplementary Table S2) and male (Supplementary Table S3). For men were observed a larger number of variables (VLF, LF and HF) with significant associations in both ETTs. Females presented significant associations only during T1, in stages 3 and 4 for HF and in stages 2 and 3 for VLF.

## Discussion

In this research, we demonstrated that there are differences in autonomic and hemodynamic responses during ETT performed with and without handrail support.

In the current investigation, measures of SBP followed an exponential growth during the ETT, as expected with rising exercise intensity^19^. In T2, SBP reached higher values for more advanced stages of ETT compared to the same stages in T1, proving the superior cardiovascular demand when not holding onto the handrails. However, DBP remained stable in both tests but reached higher values in T1. Studies have shown that DBP tends to remain constant, as a result of the balance between muscle vasodilatation, which decreases peripheral resistance, and vasoconstriction in areas not used during exercise^19–21^. The BP behavior seems to be related to the reflex response of the cardiac autonomic modulation, which is stimulated by the activation of mechanoreceptors in the musculature^22^. In addition, studies revealed that DBP is highly associated with peripheral resistance, and even a small change in this variable can result in oscillation on DBP^19,22^. Based on previous studies^19,22^, our results suggest that not holding on the handrails (T2) increased vasodilation in exercise muscle due to superior exercise intensity, and, as a result DPB and peripheral resistance were reduced.

In T2, we observed lower values for VO_2_ and HRV components. The estimated VO_2_ is an important parameter in the evaluation of aerobic capacity, and it is closely related to the cardiac output and time elapsed on the ETT*.* The difference of more than 20% in VO_2_ between T1 and T2 might be associated with the way that the tests were performed. As the duration of T2 tests was shorter, we concluded that the maximum physical capacity was reached more quickly, consequently decreasing the VO_2_ values.

Previous studies have already found that the incremental growth in exercise intensity has a positive effect on HR and, consequently, HRV decreases^16,22–26^. This response is associated with progressive inhibition of the parasympathetic pathway that occurs until it reaches the ventilatory threshold, when it disappears^16,22–27^. Shiraishi et al.^16^ and Karapetian et al.^27^ verified that there is a strong relation between the ventilatory threshold and the change that occurs in HF of the HRV in the transition from aerobic to anaerobic metabolism.

Hence, as HR in T2 presented itself higher than in T1 throughout the ETT, it corroborated the hypothesis that the individual exerts a greater effort when not holding onto the handrails of the treadmill. Moreover, in the early stages, there was a difference of almost 30% between the decrease in the HF band, frequency spectrum associated with vagal activity, in the different ETT performed^23^. One of the explanations suggested for the difference found in HF in the early stages (S1, S2 S3) can be explained by the considerations of Arai et al.^23^, that the greatest decrease in vagal activity in T2 when compared with T1 is due to the greater possibility of response to respiratory fluctuation when you do not hold on to the treadmill.

Furthermore, when the subject is above the ventilatory threshold, the respiratory rate does considerable changes to the end of the ETT^28^. Other notable study^16^ have found that in healthy volunteers and patients with myocardial infarctions when HF was absent it corresponded to the ventilatory threshold. Therefore, it can be assumed that these changes in the HF spectral component and pNN50 during alterations of the breathing frequency are either because of non-parasympathetic influences, or, these methods of analysis of cardiac parasympathetic tone may be biased by the changing respiratory pattern.

Shiraishi et al.^16^ demonstrated that HF index corresponded to the ventilatory threshold. In addition to presenting higher estimated VO_2_ values at the beginning of the ETT, the HR was still under parasympathetic regulation. Thus, the possible reduction of the physical load in the test performed incorrectly made the exercise more moderate, meaning that more time was needed to reach the ventilatory threshold. Therefore, the parasympathetic pathway remained active for a longer period.

In findings related to LF, we found an association with VO_2_ in more stages of T1 than T2. This fact highlights the influence that baroreflex activity, represented by LF^29^, has in cardiovascular adjustments to exercise. In a study carried out with an animal model, it was verified that the sympathetic activity became the modulating agent in the elevation of the HR when the duration or intensity of the exercise were increased^30^. Paterson^31^ showed a mechanism in which demonstrated that autonomic modulation would be dependent on nitric oxide (NO) receptors. Furthermore, NO seems to be involved in regulation of baroreceptor neurons and these receptors interfere in the modulation of muscle sympathetic nerve activity response during exercise^32^. In this context, Fisher et al.^33^ reviewed the autonomic adjustments that occur during exercise. They summarized that autonomic modulation is highly related to exercise intensity and it involves several mechanisms, such as central command, baroreceptors, arterial baroreflex, chemoreceptors and respiratory rate. Also, the progressive decrease in cardiac parasympathetic activity is the most responsible for HR response during exercise. According to the article^33^, corroborating our findings, when performing an incremental exercise with extensive muscle workload, HR and oxygen uptake (VO_2_) had a linear association. Thus, we can infer that when used handrail support (T1), the volunteer reached the ventilatory threshold later than in T2 and as a result, the parasympathetic influences on HR were slightly prolonged during T1.

The supplementary results showed more expressive association between VO_2_ and all variables of autonomic function for both ETTs, specially for men. A possible explanation for this difference could be based on Lundsgaard et al.^34^ review, in which highlighted that VO_2_ peak is apparently 10–20% higher in men than women due to larger muscle mass and higher hemoglobin concentration. Furthermore, Fisher et al.^33^ accounted that autonomic modulation depends on the feedback of mechanical and metabolic sensitive afferent fibers from skeletal muscle to the exercise intensity.

In summary handrails support during the ETT significantly influences in the estimative of cardiorespiratory fitness and autonomic function, specially on parasympathetic pathway.

### Study limitations

Nonetheless, the study has some limitations. The BP measurement may have been compromised, since it was evaluated with the volunteer in motion. In addition, there is no control over the participant's lifestyle. This is due to the fact that habits such as sleep deprivation and excessive food consumption, which have an influence on autonomic activity, are unknown. These characteristics and the non-identification of associated morbidities, which were not evaluated and therefore have an undisclosed presence, could possibly affect the results. For results analysis, we used the averages of the predetermined intervals. Therefore, the estimation of data values may be limited due to their interpretation. If we observe the dynamics of the autonomic function variables in real time, as reported in the study by Shiraishi et al.^16^, there will be a greater accuracy of results when each aspect is analyzed separately. Also considering autonomic function, it's important to be cautious about the use and interpretation of information of the relationship between the HF and parasympathetic nervous activity during exercise testing^35^.

### Perspective

The study has demonstrated that using handrail support during an ergometric test will result in an overestimation of aerobic capacity and an artificial increase on the length of the test. It would interfere on interpretation of diagnosis, prognosis and functional capacity of the patient analyzed, as they all depend on the duration of the test. The recommendation for situations in which the patient requires or has a condition that demands him/her to use handrail support is to register this information and to interpret the results with variances that will occur with this specific performance, and also standardize it. The results are aligned with the AHA recommendation that holding on to the front and side handrails can reduce body load, with subsequent reduction of physical exertion, hence underestimating physical and hemodynamic responses.

To summarize, the exercise treadmill testing is a path to verifying an individual's physical capacity and their cardiovascular responses to physical effort. By measuring these responses through HRV, it is possible to verify the difference between effort tests performed in correct and incorrect positioning.

Therefore, confirming the hypothesis raised, we concluded that, in the execution of the ETT without holding on to the treadmill, the volunteer/subject’s performance is in agreement with the reality of the effort made. The parasympathetic component influences in HR were slightly prolonged when the ETT is performed incorrectly, that is, the individual reaches his/her maximum effort later, overestimating it. Nevertheless, it is necessary to standardize positioning during the execution of the ETT individually, in order to avoid inadequate interpretation of the results observed during and in the end of the test.

## Methods

### Study design and participants

The development of this research was based on a cross-sectional descriptive study^36^. with a simple random sampling of students between 20 and 25 years old, enrolled in Health-related majors at the Federal University of Paraná (UFPR) in Curitiba, Brazil. Volunteers were asked to make a battery of evaluations on two nonconsecutive days. They could withdraw from participating in the survey at any time. All data was collected in the afternoon. Temperature and humidity were not standardized because there was no possibility of having a controlled environment in our facilities.

On the first day, each participant was submitted to the following evaluations: anamnesis, anthropometry measurements and HRV at rest and during Exercise Treadmill Testing (ETT), when use of handrail support during ETT. Upon returning after 7 days, the same volunteer underwent another ETT, this time in the position recommended by AHA^11^.

During both tests the dynamic cardiac autonomic response of the participants was verified through their HRV.

### Anthropometric measurements

Anthropometric variables were used to characterize the sample. Volunteers had body mass (kg), standing height (cm) and waist circumference (cm) measured.

Body mass was measured using an electronic anthropometric scale (WCS^®^), with 0.1-kg resolution. All subjects were evaluated standing barefoot, in light clothing^17^.

Height was measured using a wall-mounted stadiometer (Wiso), with 0.1-cm resolution. Volunteers were evaluated standing barefoot on the base of the stadiometer, forming a right angle with the vertical edge of the device^17^. Some anatomical points of reference were verified during measurement, such as heels together, arms hanging freely on the sides with palms facing the thighs, and centered head in the Frankfurt position^17^. After collecting these data, the Body Mass Index (BMI) was calculated and classified.

Waist circumference (WC) was measured at the midpoint between the last costal arch and the upper arch of the iliac crest, using a SANNY flexible tape with 0.1 cm measurement resolution^17^. WC was classified as follows: normal (< 90 cm for men and < 83 cm for women); relative risk (≥ 90 cm men and ≥ 83 cm for women); absolute risk (≥ 100 cm for men and ≥ 93 cm for women)^18^.

### Heart rate variability

The autonomic system modulation of the cardiac muscle was evaluated by measuring the heart rate variability (HRV) indexes at rest and during the ergometric tests. Heart rate and one RR interval intervals were monitored and recorded every second on the heart monitor (Polar^®^ V800). Previous studies have reported the validity of Polar monitors, specially V800, as an instrument for measuring HRV during exercise and rest. The RR intervals are highly consistent with ECG measures^37–39^.

The equipment was placed on the volunteers’ precordium region and fastened to their backs by a belt with an elastic system. The recordings were then transferred to a computer, and the HRV was analyzed using the Polar Flow software for V800 CX. Volunteers’ heartbeats were recorded at rest in the supine position on a comfortable stretcher. They remained in that position for 10 min, in a quiet, air-conditioned room, in the afternoon. In the ST, HRV was recorded at all stages of the protocol, as well as at rest.

The variables related to HRV were observed by linear methods divided into time domain analysis performed using statistical and geometric indexes, and frequency domain analysis. According to the *Task Force of the European Society of Cardiology* (TFESC), the *North American Society of Pacing and Electrophysiology*^39^, the results of the HRV analysis in the time domain are expressed in milliseconds (ms). In this case, each normal RR interval (sinus beats) is measured over a given time interval. Based on statistical or geometric methods (average, standard deviation and indexes derived from the histogram or Cartesian coordinate map of the RR intervals), the fluctuations indexes during the cardiac cycles are calculated.

The records were demonstrated by the Standard deviation of normal-to-normal RR intervals SDNN (Standard deviation of normal-to-normal RR intervals), SDANN (Standard deviation of the averages of RR intervals in 5-min segments), and SDNNi (Mean of the standard deviations of RR intervals in 5-min segments) indexes and represent the sympathetic and parasympathetic activities. The rMSSD (Root mean square of successive RR interval differences) and pNN50 (Relative number of successive RR interval pairs that differ more than 50 ms) indexes represent the parasympathetic activity since they were found from the analysis of adjacent RR intervals^39,40^. The high frequency (HF) component corresponds to respiratory modulation and is an indicative of the vagus nerve acting on the heart; the low frequency (LF) component provides an index of baroreflex function^28^ and very low frequency (VLF) component is apparently related to the renin–angiotensin–aldosterone system, thermoregulation, and peripheral vasomotor tone^41^. Therefore, the LF/HF relationship was not considered for analysis as it seems to be controversial as a sympathovagal balance measure^29,39,42^.

Variables in the time domain were not considered for the analysis of each stage, because according to Malik et al.^41^, this measure must be considered when evaluating HRV in more than 5 min. As each stage of the exercise test protocol has 3 min, this variable was not considered for analysis.

### Exercise treadmill testing (ETT) with *Ellestad* protocol

The ergometric ETTs were performed on treadmill (ATL, Imbramed, Porto Alegre, Brazil) to evaluate the dynamics of HRV. The process comprised two steps. First, the volunteer was asked to hold on to the handrails of the treadmill (T1). In the second step, performed seven days after the first one, the volunteer was asked not to rest or lean on the handrails during the new test (T2). The order of tests was not randomized due to the logistics to a better adherence of the participants on the research. Yet, all participants went through the same procedure of data collecting. In both tests (T1 and T2) the blood pressure (BP) was measured before beginning of the test (baseline) and during the test in the end of each stage.

Following the *Ellestad* protocol, HR was monitored on the cardiac monitor in baseline and throughout the test (Polar V800cx). The tests took place in the afternoon, comprising progressive stages of 2 min each, besides stage one (S1) and stage five (S5) that length 3 min each. The initial stage (S1) speed was 2.7 km/h with a 10% inclination. Inclination remained constant (10%) during the first four stages (S1–S4). From stage five (S5), it raised to 15% and endured until the end of the test. In S2 speed was 4.8 km/h; in S3 it increased to 6.8 km/h; in S4 and S5 was 8 km/h; stage six (S6) was 9.5 km/h and stage 7 (S7) was 11.3 km/h^28^. Following peak exercise, the subjects walked for a three-minute cool-down period at 2.5 km/h and no inclination^28^. The tests were discontinued when the subject reported any discomfort or clinical symptoms, such as fatigue and dyspnea. Individuals who could not finish the test or did not perform cool-down period for any reason were not included in the analysis. VO_2_ was calculated after the ETT according to ACSM's guidelines for exercise testing and prescription^28^.

### Statistical analysis

Initially, for statistical analysis, we performed a normality test (Smirnov Kolmogorov), followed by a descriptive analysis of the data. This analysis was carried out through central tendency measures with mean and standard deviation (SD) for the continuous variables. We also performed frequency analysis (relative and absolute percentage) for categorical variables.

In order to verify the difference between the methods of application in the ST, *Student*’s *t*-test was used for paired data, hemodynamic measures (SBP, DBP, FC, and VO_2_). Then, the mean hemodynamic responses at each stage were analyzed in each ST method and compared using a *two-way* ANOVA. To the detriment of the hemodynamic responses and based on the hypotheses formulated, the responses of the autonomic function were also observed through the generated variables. In this case, the difference in behavior of these responses at rest was verified, T1 and T2 based on the *one-way* ANOVA. As a supplementary analysis, the same statistical test was performed for each sex separately. In addition, for each stage of the test, the mean autonomic response of each variable (frequency domain) was obtained using the *two-way* ANOVA.

At last, an Ordinal Multinomial Linear Regression analysis was performed with the *Backward for Wald method.* In this analysis, each stage of the test was considered a model, VO_2_ was considered the dependent variable, and the autonomic function variables (VLF, LF, HF) were considered the independent variable (factors). As a supplementary analysis, the same statistical test was performed for each sex separately. All analyzes were performed using SPSS for *Windows*, version 21.0, considering p < 0.05. HRV was determined using the Kubios software. A power of 90% was adopted for a two-tailed test and a maximum allowed error of 1%. The calculation was estimated at 21 subjects per group.

### Ethical approval

Written and informed consent was obtained prior to performing any testing. This study was conducted at UFPR’s School Unit of Health Promotion (SU-HP/UFPR) and approved by the Federal University of Paraná’s Research Ethics Committee (CAAE, 71645617.4.0000.0102). All testing procedures were conducted in accordance to the Declaration of Helsinki, with the exception of registration in a database.

## Supplementary information

Supplementary Information

## References

[1] Santos, T. S. N. P. *et al.* in *Cardiologia do Exercício: Do Atleta ao Cardiopata* (eds CE NEGRÃO & ACP BARRETTO) (2010).

[2] Aubert AE, Seps B, Beckers F (2003). Heart rate variability in athletes. Sports Med..

[3] Aeschbacher S (2016). Healthy lifestyle and heart rate variability in young adults. Eur. J. Prev. Cardiol..

[4] Task Force of The European Society of Cardiology and The North American Society of Pacing and Electrophysiology (1996). Heart rate variability. Standards of measurement, physiological interpretation, and clinical use. Eur. Heart J..

[5] O'Sullivan SE, Bell C (2000). The effects of exercise and training on human cardiovascular reflex control. J. Auton. Nerv. Syst..

[6] Vanderlei LCM, Pastre CM, Hoshi RA, Carvalho TDD, Godoy MFD (2009). Noções básicas de variabilidade da frequência cardíaca e sua aplicabilidade clínica. Braz. J. Cardiovasc. Surg..

[7] Castro RRTD, Lima SP, Sales ARK, Nóbrega ACLD (2017). Minute-ventilation variability during cardiopulmonary exercise test is higher in sedentary men than in athletes. Arquivos Bras. de Cardiol..

[8] Stein PK, Barzilay JI, Chaves PH, Domitrovich PP, Gottdiener JS (2009). Heart rate variability and its changes over 5 years in older adults. Age Ageing.

[9] Tulppo MP, Makikallio TH, Seppanen T, Laukkanen RT, Huikuri HV (1998). Vagal modulation of heart rate during exercise: Effects of age and physical fitness. Am. J. Physiol..

[10] La Rovere MT (2003). Short-term heart rate variability strongly predicts sudden cardiac death in chronic heart failure patients. Circulation.

[11] Fletcher GF (2001). Exercise standards for testing and training: A statement for healthcare professionals from the American Heart Association. Circulation.

[12] Leicht AS, Sinclair WH, Spinks WL (2008). Effect of exercise mode on heart rate variability during steady state exercise. Eur. J. Appl. Physiol..

[13] Martinmaki K, Rusko H (2008). Time-frequency analysis of heart rate variability during immediate recovery from low and high intensity exercise. Eur. J. Appl. Physiol..

[14] Kaikkonen P, Nummela A, Rusko H (2007). Heart rate variability dynamics during early recovery after different endurance exercises. Eur. J. Appl. Physiol..

[15] Neto VGC, Bentes CM, Neto GDAM, Miranda H (2017). Hipotensão e variabilidade da frequência cardíaca pós-exercício de força executado de forma máxima e submáxima. Motricidade.

[16] Shiraishi Y (2018). Real-time analysis of the heart rate variability during incremental exercise for the detection of the ventilatory threshold. J. Am. Heart Assoc..

[17] Crawford, S. M. in *Measurement in pediatric exercise science* (ed Docherty, D.) 18–86 (Human Kinetics, 1996).

[18] Zhu S (2002). Waist circumference and obesity-associated risk factors among whites in the third National Health and Nutrition Examination Survey: Clinical action thresholds. Am. J. Clin. Nutr..

[19] Mitchell JH, Kaufman MP, Iwamoto GA (1983). The exercise pressor reflex: Its cardiovascular effects, afferent mechanisms, and central pathways. Annu. Rev. Physiol..

[20] Secher NH, Amann M (2012). Human investigations into the exercise pressor reflex. Exp. Physiol..

[21] Monteiro MDF, Sobral Filho DC (2004). Exercício físico e o controle da pressão arterial. Rev. Bras. de Med. do Esporte.

[22] Raven PB (2008). Recent advances in baroreflex control of blood pressure during exercise in humans: An overview. Med. Sci. Sports. Exerc..

[23] Arai Y (1989). Modulation of cardiac autonomic activity during and immediately after exercise. Am. J. Physiol..

[24] Warren JH, Jaffe RS, Wraa CE, Stebbins CL (1997). Effect of autonomic blockade on power spectrum of heart rate variability during exercise. Am. J. Physiol..

[25] Shibata M, Moritani T, Miyawaki T, Hayashi T, Nakao K (2002). Exercise prescription based upon cardiac vagal activity for middle-aged obese women. Int. J. Obes. Relat. Metab. Disord..

[26] Carter JB, Banister EW, Blaber AP (2003). Effect of endurance exercise on autonomic control of heart rate. Sports Med..

[27] Karapetian GK, Engels HJ, Gretebeck RJ (2008). Use of heart rate variability to estimate LT and VT. Int. J. Sports Med..

[28] Riebe D, Ehrman JK, Liguori G, Magal M, American College of Sports Medicine (2018). ACSM's Guidelines for Exercise Testing and Prescription.

[29] Goldstein DS, Bentho O, Park MY, Sharabi Y (2011). Low-frequency power of heart rate variability is not a measure of cardiac sympathetic tone but may be a measure of modulation of cardiac autonomic outflows by baroreflexes. Exp. Physiol..

[30] Negrao CE, Moreira ED, Brum PC, Denadai ML, Krieger EM (1992). Vagal and sympathetic control of heart rate during exercise by sedentary and exercise-trained rats. Braz. J. Med. Biol. Res..

[31] Paterson D (2001). Nitric oxide and the autonomic regulation of cardiac excitability. The G.L. Brown prize lecture. Exp. Physiol..

[32] Raven P, Young B, Fadel P (2019). Arterial baroreflex resetting during exercise in humans: Underlying signaling mechanisms. Exerc. Sport Sci. Rev..

[33] Fisher JP, Young CN, Fadel PJ (2015). Autonomic adjustments to exercise in humans. Compr. Physiol..

[34] Lundsgaard, A.-M., Fritzen, A. M., & Kiens, B. Exercise physiology in men and women. in *Principles of Gender-Specific Medicine*, 525–542 (2017).

[35] Katona Peter G, Jih FE (1975). Respiratory sinus arrhythmia: noninvasive measure of parasympathetic cardiac control. J. Appl. Physiol..

[36] Thomas JR, Nelson JK, Silverman SJ (2015). Research Methods in Physical Activity.

[37] Caminal P (2018). Validity of the Polar V800 monitor for measuring heart rate variability in mountain running route conditions. Eur. J. Appl. Physiol..

[38] Giles D, Draper N, Neil W (2016). Validity of the Polar V800 heart rate monitor to measure RR intervals at rest. Eur. J. Appl. Physiol..

[39] Task Force of the European Society of Cardiology the North American Society of Pacing Electrophysiology (1996). Heart rate variability. Standards of measurement, physiological interpretation, and clinical use. Eur. Heart J..

[40] Novais LD (2004). Avaliação da variabilidade da frequência cardíaca em repouso de homens saudáveis sedentários e de hipertensos e coronariopatas em treinamento físico. Rev. Bras. de Fisioter..

[41] Malik M, Bigger JT, Camm AJ, Kleiger RE, Malliani A, Moss AJ, Schwartz PJ (1996). Heart rate variability: Standards of measurement, physiological interpretation, and clinical use. Eur. Heart J..

[42] Stebbings GK, Morse CI, McMahon GE, Onambele GL (2013). Resting arterial diameter and blood flow changes with resistance training and detraining in healthy young individuals. J. Athl. Train..

