# Author Correction: Handrail support interference in cardiac autonomic modulation adjustments in young adults during maximal exercise testing

**DOI:** 10.1038/s41598-020-75176-5

**Published:** 2020-10-28

**Authors:** Giovanna Lima de Oliveira, Adriana Hernandez Marques, Vanessa Ferrari da Fonseca, Beatriz Augusta Pozzolo, Fernanda Panacioni, Taís Capucho Santos, Amanda Archeleiga Guedes, Aurenzo Gonçalves Mocelin, Renata Labronici Bertin, Anderson Zampier Ulbrich

**Affiliations:** 1 Research Group of Exercise Medicine (MedEx), Federal University of Parana (UFPR), Padre Camargo Street, 280– Alto da Gloria, Curitiba, PR 80060‑240 Brazil; 2 Department of Nutrition, Center for Health Sciences, Federal University of Parana (UFPR), Curitiba, PR Brazil; 3 Department of Integrative Medicine, Center for Health Sciences, Federal University of Parana (UFPR), Curitiba, PR Brazil

Correction to: *Scientific Reports* 10.1038/s41598-020-68155-3, published online 08 July 2020

The original version of this Article contained a typographical error in the spelling of the author Amanda Archeleiga Guedes, which was incorrectly given as Amanda Acheleiga Guedes. This has now been corrected in the PDF and HTML versions of the Article, and in the accompanying Supplementary Information file.

