# Supplementary material for: Handrail support interference in cardiac autonomic modulation adjustments in young adults during maximal exercise testing
Source: Sci Rep. 2020 Jul 8;10:11196. doi: 10.1038/s41598-020-68155-3 (PMC7343795; doi:10.1038/s41598-020-68155-3)
Supplement: Supplementary file 1 — Supplementary Information [file 41598_2020_68155_MOESM1_ESM.docx]

# **Supplementary information**

# **Handrail support interference in Cardiac Autonomic Modulation Adjustments in young adults during maximal exercise testing**

Giovanna Lima de Oliveira^1^, Adriana Hernandez Marques^1^, Vanessa Ferrari da Fonseca^1^, Beatriz Augusta Pozzolo^1^, Fernanda Panacioni^1^, Taís Capucho Santos^1^, Amanda Archeleiga Guedes^1^, Aurenzo Gonçalves Mocelin^1^, Renata Labronici Bertin^1,2^, Anderson Zampier Ulbrich^1,3^

^1^ Federal University of Parana (UFPR), Curitiba, PR, Brazil. Research Group of Exercise Medicine (MedEx).

^2^ Department of Nutrition, Center for Health Sciences, Federal University of Parana (UFPR), Curitiba, PR, Brazil.

^3^ Department of Integrative Medicine, Center for Health Sciences, Federal University of Parana (UFPR), Curitiba, PR, Brazil.

**Corresponding author:**

Anderson Zampier Ulbrich

Padre Camargo Street, 280 – Alto da Gloria - Curitiba – PR. Zip Code: 80.060-240 e-mail: anderon_u@hotmail.com or medexufpr@gmail.com

Phone: +55 xx 41 3360-7228

**Table S1.** Overall evaluation for both sexes, disregarding the stages, of the HRV variables at rest, holding (T1) and not holding (T2) on to the handrails of the treadmill.

|  |  |  | **Rest** | **T1** | **T2** | ***P* value^#^** |
| --- | --- | --- | --- | --- | --- | --- |
| **Female** | Time domain | **RR** | 789.40 ± 79.25 | 370.59 ± 101.59 | 400.50 ± 24.88 | 0.275 |
|  |  | **SDNN** | 49.70 ± 17.76 | 10.90 ± 7.92 | 11.09 ± 11.90 | 0.960 |
|  |  | **RMSSD** | 52.32 ± 25.30 | 9.78 ± 8.55 | 10.19 ± 9.34 | 0.891 |
|  |  | **NNxx** | 193.37 ± 109.96 | 11.00 ± 17.45 | 3,37 ± 3,68* | 0.108 |
|  | Frequency Domain | **VLF, ms^2^** | 152.50 ± 104.68 | 10.87 ± 7.41 | 11.00 ± 10.39 | 0.968 |
|  |  | **HF, ms^2^** | 1282.68 ± 1255.73 | 27.68 ± 33.08 | 16.56± 22.98 | 0.153 |
|  |  | **LF, ms^2^** | 1063.56 ± 821.46 | 60.18 ± 51.02 | 40.50 ± 36.24 | 0.086 |
|  |  |  | **Rest** | **T1** | **T2** | ***P* value^#^** |
| Male | Time domain | **RR** | 873.05 ± 104.68 | 398.38 ± 26.83 | 393.68 ± 22.00 | 0.482 |
|  |  | **SDNN** | 53.71 ± 23.67 | 9.84 ± 4.59 | 9.36 ± 3.55 | 0.643 |
|  |  | **RMSSD** | 52.60 ± 28.22 | 8.98 ± 5.43 | 8.03 ± 4.48 | 0.423 |
|  |  | **NNxx** | 164.26 ± 105.43 | 10.52 ± 12.17 | 3,57 ± 3,97* | 0.015 |
|  | Frequency Domain | **VLF, ms^2^** | 196.05 ± 175.13 | 12.84 ± 12.17 | 14.94 ± 12.95 | 0.584 |
|  |  | **HF, ms^2^** | 1307.94 ± 1384.30 | 47.21 ± 62.36 | 26.57± 30.81* | 0.145 |
|  |  | **LF, ms^2^** | 1566.52 ± 1386.99 | 71.00 ± 62.17 | 67.52 ± 55.30 | 0.823 |

#paired comparison between T1 and T2;

Values reported in mean ± standard deviation; RR: RR interval; SDNN: standard deviation of all normal RR intervals; RMSSD: root mean square of successive squared differences between adjacent normal RRs; NNxx: number of interval differences of successive intervals NN greater than xx = ^39 40, 50^ ms; VLF: very low frequency component; HF: high frequency component; LF: low frequency component, LF/HF: ratio between LF and HF. **P* <0.05.

**Table S2**. Association of VO2 with the components of HRV determined in the stages of the different ETT. (FEMALE)

| **T1** | | | | | | | | | | | | | |
| --- | --- | --- | --- | --- | --- | --- | --- | --- | --- | --- | --- | --- | --- |
|  | **S1**  **β (95% CI)** | ***P* value** | **S2**  **β (95% CI)** | ***P* value** | **S3**  **β (95% CI)** | ***P* value** | **S4**  **β (95% CI)** | ***P* value** | **S5**  **β (95% CI)** | ***P* value** | **REC**  **β (95% CI)** | ***P* value** |  |
| **VLF** | 0.115  (-0.036; -0.266) | 0.123 | **0.589**  (0.085; 1.092) | **0.025*** | **1.738**  (0.040; 3.437) | **0.046*** | 1.814  (-6.160; 9.789) | 0.614 | 3.586  (-3.548; 6.720) | 0.506 | **-3.870**  (-6.192 -1.549) | **0.003*** |  |
| **LF** | -1.179  (-3.074; -0.717) | 0.202 | -0.168  (-0.385; -0.049) | 0.119 | 0.150  (1.261; 1.561) | 0.821 | -6.245  (-10.081; -2.408) | 0.005* | 5.191  (-3.222; 12.602) | 0.531 | **1.354**  (0.233; 2.476) | **0.022*** |  |
| **HF** | -0.057  (-0.161; -0.047) | 0.259 | -0.027  (-0.184; -0.129) | 0.707 | **1.012**  (0.224; 1.799) | **0.016*** | **1.821**  (0.973; 2.669) | **0.001*** | 1.795  (-8.646; 12.236) | 0.658 | 0.093  (-0.137; 0.323) | 0.392 |  |
| **T2** | | | | | | | | | | | | | |
| **VLF** | 0.006  (-0.211; 0.213) | 0.948 | 0.551  (-1.231; 0.128) | 0.103 | -0.180  (-2.466; 2.105) | 0.865 | **2.015**  (1.318; 4.712) | **0.029*** | - | - | **-0.161**  (-0.297; -0.025) | **0.024*** |  |
| **LF** | 0.021  (-0.006; 0.048) | 0.113 | 0.051  (-0.074; 0.176) | 0.393 | -0.273  ( -0.703; 0.156) | 0.194 | -1.341  (-2.741; 5.422) | 0.437 | - | - | 0.085  (-0.171; 0.341) | 0.483 |  |
| **HF** | -0.019  (-0.077; 0.040) | 0.504 | 0.021  (-0.152; 0.194) | 0.795 | 0.166  (-0.856; 1.189) | 0.729 | -4.745  (-9.726; 0.237) | 0.059 | - | - | -0.042  (-0.304; 0.219) | 0.728 |  |

Values reported in calculated value of β (minimum value - maximum value). The value of β corresponds to how many ml.kg^-1^.min^-1^ are added to VO_2_ when a unit of the HRV variable is increased.  The analysis was performed only including values from S1 to S5 because the number of subjects who participate of S6 and S7 were not sufficient to obtain statistically significant results. VLF: very low frequency component; HF: high frequency component; LF: low frequency component, L/H: ratio between LF and HF; REC to recovery period of the test (three minutes); S1: stage one; S2: stage two; S3: stage three; S4: stage four; S5: stage five; REC: recovery time to ST with a three-minute duration. **P*<0.05**.**

T**able S3**. Association of VO2 with the components of HRV determined in the stages of the different ETT. (MALE)

| **T1** | | | | | | | | | | | | |
| --- | --- | --- | --- | --- | --- | --- | --- | --- | --- | --- | --- | --- |
|  | **S1**  **β (95% CI)** | ***P* value** | **S2**  **β (95% CI)** | ***P* value** | **S3**  **β (95% CI)** | ***P* value** | **S4**  **β (95% CI)** | ***P* value** | **S5**  **β (95% CI)** | ***P* value** | **REC**  **β (95% CI)** | ***P* value** |
| **VLF** | 0.026  (-0.099; 0.151) | 0.666 | -0.113  (-0.723; 0.496) | 0.696 | **-1.474**  (-2.681; -0.267) | **0.020*** | **3.502**  (0.107; 6.896) | **0.044*** | -3.346  (-21.476; 14.784) | 0.692 | -0.172  (-1.090; 1.433) | 0.775 |
| **LF** | 0.008  (-0.006; 0.022) | 0.226 | **0.055**  (0.003; 0.107) | **0.039*** | **0.929**  (0.330; 1.529) | **0.005*** | 1.278  (-2.286; 4.842) | 0.455 | -0.658  (-1.709; 0.393) | 0.199 | -0.647  (-1.728; 0.434) | 0.223 |
| **HF** | -0.006  (-0.067; 0.055) | 0.837 | **-0.047**  (-0.155; -0.006) | **0.049*** | **-0.639**  (-1.088; -0.190) | **0.008*** | 1.389  (-0.629; 3.407) | 0.163 | 1.619  (-0.001; 3.235) | 0.051 | 1.482  (-1.165; 4.130) | 0.254 |
| **T2** | | | | | | | | | | | | |
| **VLF** | 0.008  (-0.040; 0.055) | 0.726 | 0.036  (-0.288; 0.359) | 0.818 | 0.204  (-1.614; 2.023) | 0.813 | -0.062  (-3.623; 3.499) | 0.971 | 2.096  (-7.625; 11.816) | 0.626 | 0.240  (-1.034; 1.515) | 0.695 |
| **LF** | 0.004  (-0.004; 0.011) | 0.347 | **0.065**  (0.024; 0.107) | **0.004*** | **0.758**  (0.171; 1.345) | **0.015*** | -0.192  (-0.921; 0.537) | 0.583 | -1.098  (-5.084; 2.888) | 0.543 | - 0.227  (- 0.863; 0.409) | 0.457 |
| **HF** | -0.013  (-0.040; 0.014) | 0.313 | **-0.059**  (-0.113; -0.005) | **0.033*** | **-0.359**  (-0.674; -0.045) | **0.028*** | 0.196  (-0.409; 0.802) | 0.502 | 0.469  (-1.159; 2.096) | 0.531 | -0.031  (-0.171; 0.110) | 0.652 |

Values reported in calculated value of β (minimum value - maximum value). The value of β corresponds to how many ml.kg^-1^.min^-1^ are added to VO_2_ when a unit of the HRV variable is increased.  The analysis was performed only including values from S1 to S5 because the number of subjects who participate of S6 and S7 were not sufficient to obtain statistically significant results. VLF: very low frequency component; HF: high frequency component; LF: low frequency component, L/H: ratio between LF and HF; REC to recovery period of the test (three minutes); S1: stage one; S2: stage two; S3: stage three; S4: stage four; S5: stage five; REC: recovery time to ST with a three-minute duration. **P*<0.05**.**
